# Early Mobilization After Stroke: Do Clinical Practice Guidelines Support Clinicians' Decision-Making?

**DOI:** 10.3389/fneur.2021.606525

**Published:** 2021-02-05

**Authors:** Venesha Rethnam, Kathryn S. Hayward, Julie Bernhardt, Leonid Churilov

**Affiliations:** ^1^Florey Institute of Neuroscience and Mental Health, Heidelberg, VIC, Australia; ^2^National Health and Medical Research Council (NHMRC) Centre for Research Excellence in Stroke Rehabilitation and Brain Recovery, Melbourne, VIC, Australia; ^3^Melbourne School of Health Sciences, University of Melbourne, Parkville, VIC, Australia; ^4^Melbourne Medical School, University of Melbourne, Parkville, VIC, Australia; ^5^Melbourne Brain Centre, Royal Melbourne Hospital, Melbourne, VIC, Australia

**Keywords:** clinical practice guidelines, stroke, AGREE-REX, acute stroke the limitations of early mobilization clinical practice guidelines, agree, early mobilization

## Abstract

**Importance:** Early mobilization, out-of-bed activity, is a component of acute stroke unit care; however, stroke patient heterogeneity requires complex decision-making. Clinically credible and applicable CPGs are needed to support and optimize the delivery of care. In this study, we are specifically exploring the role of clinical practice guidelines to support individual patient-level decision-making by stroke clinicians about early mobilization post-stroke.

**Methods:** Our study uses a novel, two-pronged approach. (1) A review of CPGs containing recommendations for early mobilization practices published after 2015 was appraised using purposely selected items from the Appraisal of Guidelines Research and Evaluation–Recommendations Excellence (AGREE-REX) tool relevant to decision-making for clinicians. (2) A cross-sectional study involving semi-structured interviews with Australian expert stroke clinicians representing content experts and CPG target users. Every CPG was independently assessed against the AGREE-REX standard by two reviewers. Expert stroke clinicians, invited via email, were recruited between June 2019 to March 2020.The main outcomes from the review was the proportion of criteria addressed for each AGREE-REX item by individual and all CPG(s). The main cross-sectional outcomes were the distributions of stroke clinicians' responses about the utility of CPGs, specific areas of uncertainty in early mobilization decision-making, and suggested parameters for inclusion in future early mobilization CPGs.

**Results:** In 18 identified CPGs, many did not adequately address the “Evidence” and “*Applicability to Patients”* AGREE-REX items. Out of 30 expert stroke clinicians (11 physicians [37%], 11 physiotherapists [37%], 8 nurses [26%]; median [IQR] years of experience, 14 [10–25]), 47% found current CPGs “too broad or vague,” while 40% rely on individual clinical judgement and interpretation of the evidence to select an *evidence-based* choice of action. The areas of uncertainty in decision-making revealed four key suggestions: (1) more granular descriptions of patient and stroke characteristics for appropriate tailoring of decisions, (2) clear statements about when clinical flexibility is appropriate, (3) detailed description of the intervention dose, and (4) physical assessment criteria including safety parameters.

**Conclusions:** The lack of specificity, clinical applicability, and adaptability of current CPGs to effectively respond to the heterogeneous clinical stroke context has provided a clear direction for improvement.

## Introduction

Clinical decision-making is thought to be a contextual, continuous, and evolving process, where data are gathered, evaluated, and interpreted to select an *evidence-based* choice of action (1). The definition emphasizes the importance of both *evidence generation* and *evaluation*, and the *interpretation* that is relevant for individual patient decision-making. Clinical decisions are embedded in evidence-based medicine, which integrates clinically relevant (patient-oriented) research, clinical expertise and patient values and preferences (2, 3). However, evidence generated by randomized controlled trials (RCTs), often expressed as an average treatment effect at a population level, cannot directly support clinical decision-making at an individual patient-level (4, 5). Clinical practice guidelines (CPGs) are decision support tools that serve to fill this gap by providing recommendations, generally with consideration of clinical applicability to the patient and setting, and by explicitly stating the options for, and implication of different care options, i.e., trade-off between harm vs. benefits, to support clinical judgements (6–8).

In the domain of stroke rehabilitation and recovery, early mobilization (sitting out of bed, standing or walking early after stroke) has long been considered an important part of stoke unit care (9). CPGs have historically reflected this, and contained recommendations to mobilize patients as early as possible post-stroke (9, 10). However, a publication in 2015 of the largest RCT, A Very Early Rehabilitation Trial (AVERT; *n* = 2,104), demonstrated poorer outcomes in the early mobilization group compared to usual care (11). This resulted in many CPG recommending against [intensive] out-of-bed activity starting within 24 h post-stroke (10), with significant uncertainties about best practice care remaining. Furthermore, the recent publication of a Cochrane Review (12) and supplementary individual participant data meta-analysis (13) of early mobilization RCTs demonstrated that although early mobilization is not recommended within 24 h post-stroke, there is still a need for more detailed research to understand the optimal timing, frequency, and intensity of the intervention. This has an obvious implication for developing decision support tools.

The need for decision support stems from the significant challenges faced by stroke clinicians when translating population-level evidence from recovery and rehabilitation trials to individualized clinical decision-making. The inherent heterogeneity of stroke patients and their recovery patterns are not explicitly covered by CPG recommendations. This contributes to the complexity and ambiguity of decision-making in early mobilization practices and potential inconsistencies in the delivery of care. It is evident that clear and clinically applicable CPGs for early mobilization are required to effectively support decision-making at an individual patient level, standardize care, and ultimately optimize patient outcomes. The existing gaps in knowledge include the utility of current early mobilization CPGs to support clinical decision-making, the areas of uncertainty in decision-making, and the key recommendations to improve future early mobilization CPGs.

Our overall aim was to investigate how well-CPGs for early mobilization after stroke support individual patient-level decision-making for expert stroke clinicians. To meet this aim we:

1. Systematically evaluated how well-specific normative decision-support criteria from the Appraisal of Guidelines Research and Evaluation–Recommendations Excellence (AGREE-REX) tool are met by early mobilization CPGs.
2. Empirically investigated the influence of early mobilization CPGs on decision-making of Australian stroke clinicians', the areas of uncertainty in their decision-making, and suggestions to improve CPG recommendations.

## Materials and Methods

### Study Design

**Figure 1:**
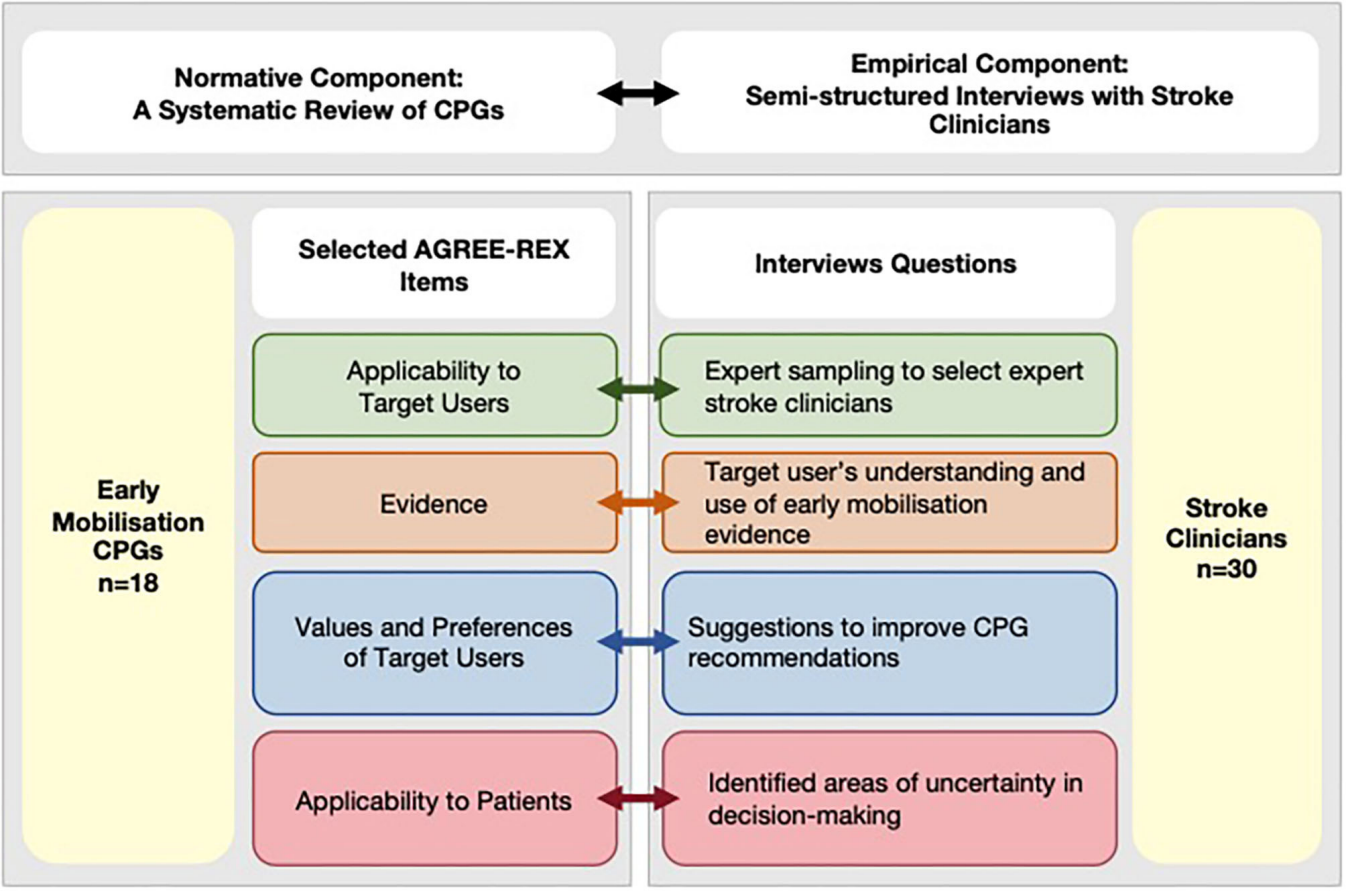
Conceptual framework integrating normative and empirical components. AGREE-REX, Appraisal of Guidelines Research and Evaluation–Recommendations Excellence; CPG, clinical practice guidelines; EM, early mobilization.

This cross-sectional study was designed using a conceptual framework developed in accordance with the definition of clinical decision-making (1) to emphasize the importance of both *evidence generation and evaluation*, as well as the *interpretation* component relevant for *individual patient decision-making* (Figure 1, top panel). For that reason, this study includes two components:

1. Normative component—a review of stroke CPGs containing recommendations for early mobilization practices. Recommendations were appraised using purposely selected AGREE-REX items that are relevant to decision-making for clinicians.
2. Empirical component—semi-structured interviews with Australian expert stroke clinicians to investigate the influence of early mobilization CPGs on decision-making of stroke clinicians', the areas of uncertainty in their decision-making, and suggestions to improve CPG recommendations.

We report this cross-sectional study in accordance with Strengthening the Reporting of Observational Studies in Epidemiology (STROBE) guidelines (14).

### Normative Component

#### Search Strategy and Data Extraction

In November 2019, the following resources were used to search for CPGs:

- Stroke CPGs identified by the World Stroke Organization (15)
- 30 early Mobilization CPGs identified by Bernhardt et al. (9)
- LMICs stroke CPGs identified by the Lancet Neurology Commission (16)
- US National Guidelines Clearinghouse
- Guidelines International Network
- Scientific Electronic Library Online (SciELO) Network
- PubMed and Excerpta Medica Database (EMBASE)
- Websites of known CPG development bodies.

Search terms included Topic = “country name” AND Topic = “guideline” OR “consensus” OR “standards” OR “recommendations” AND Topic = “stroke OR cerebrovascular disorder/disease OR intracranial hemorrhage OR cerebrovascular accident OR “early mobilization.” CPGs were selected if they were publicly available; currently active; produced with the support of a health professional association or society, public or private organization, health care organization or plan, or government agency; published after 2015 to ensure data from AVERT (11) was considered; and contained recommendations about early mobilization practices. CPGs for the management of childhood stroke were excluded. CPGs in Dutch, German, Portuguese, Russian and Ukrainian were also independently reviewed by an individual fluent in those languages. Other non-English CPGs were examined using Google Translate, which has been shown to be a viable and accurate tool for translating non–English-language trials to conduct systematic reviews (17, 18). Each CPG was reviewed and information regarding the title, year of publication, name of organization/society/government agency, specific early mobilization recommendations, evidence informing recommendations, and methodological approach was extracted for appraisal.

#### Appraisal of Clinical Practice Guidelines

The AGREE-REX (Appraisal of Guideline Research and Evaluation-Recommendations Excellence) tool was used to evaluate the quality of CPG recommendations for early mobilization post-stroke (19). The tool consists of nine items structured within three theoretical domains: clinical applicability, values and preferences, and implementability. All nine AGREE-REX items contain an operational definition of the item and several item-specific criteria (ranging between 2 and 10) for CPGs to be evaluated on.

Since the overall aim of this study was to understand the level of decision support provided in CPGs for clinicians, the four most relevant AGREE-REX items were selected for detailed review: *Evidence* (eight criteria), *Applicability to Target Users* (five criteria), *Applicability to Patients* (four criteria), and *Values and Preferences of Target Users* (three criteria). The *Evidence* and *Applicability to Patient* items of the AGREE-REX relate to the tailoring of clinical decisions to individual and/or patient subgroups. The *Applicability to Target Users* and *Values and Preferences of the Target Users* items relate to providing decision support to the stroke clinicians.

The excluded five AGREE-REX items included: *Values and Preferences of Policy/Decisionmakers, Values and Preferences of Guideline Developers, Purpose, Local Application and Adoption*, and *Values and Preferences of Patients/Population*. These items were excluded because we focused on investigating *how well-CPGs support decision-making for stroke clinicians (target user)*. Although the *Values and Preferences of Policy/Decision-makers* and *Guideline Developers* items are certainly important in developing clinically credible and implementable guidelines, these are not directly relevant to individual patient-level decision-making for stroke clinicians about early mobilization. The *Purpose* item was excluded because it was related to “the implementation goals of the guideline (e.g., for advocacy, policy change, etc.)” (19). The *Local Application and Adoption* item was excluded not to diminish the role of systems of care factors and social structures, but rather to emphasize that the four *chosen items* will be present and play a crucial role in supporting individual patient-level decision-making across different healthcare systems and social structures. The *Values and Preferences of Patients* item was excluded because we specifically focused on clinical decision-making by clinicians (target user of CPGs) rather than shared decision-making. Certainly, a future study following a similar integrated research design (i.e., collecting both normative and empirical evidence *from patients*) presents a viable future direction and could provide valuable insights into shared decision-making.

Every CPG was independently assessed against the criteria (yes/no) by two reviewers (VR, KH). The AGREE-REX items were given a score (percentage of total achievable score) depending on the number of criteria addressed by the CPG i.e., if a CPG addressed 6 out of 8 criteria for the *Evidence* item, the CPG would receive a score of 75% for that item. The following outcomes were reported: (1) a summary of AGREE-REX item scores (%) achieved by each CPG and (2) the percentage of CPGs addressing each criterion.

### Empirical Component

#### Study Participants and Recruitment

In this study, expert sampling (20) was used to recruit a representative sample of CPG target users and post-stroke early mobilization content experts (i.e., participated or conducted early mobilization research) who can provide a level of expertise necessary to understand the evidence and applicability to patient components of this study. This sample typically forms part of a guideline development group in Australia (21). As a result, we identified a sample of stroke physicians, physiotherapists and nurses/nurse practitioners with a high level of expertise in delivering acute stroke care and understanding early mobilization (senior position, >6 years stroke experience). According to this sampling strategy, clinicians identified as appropriate for participation were invited to participate via email and were recruited between June 2019 to March 2020. Ethics approval was obtained from The University of Melbourne Human Research Ethics Committee (1851680.1). Consent was obtained from all participants involved in the study.

#### Semi-structured Interview

We conducted the interviews in two distinct parts. Each addressed an individual aim: (1) to investigate the utility and limitations of current early mobilization CPGs and (2) identify the decision-making factors and specific high-interest patient subgroups using a data visualization tool. The analysis and results for the second part are presented elsewhere.

In this study, the design of the interview questions was informed by the conceptual framework that links the normative and empirical components of the study (Figure 1, bottom panel). Therefore, the topics of interest were pre-determined using selected AGREE-REX items to investigate both the *evidence generation* and *evaluation* component and the *interpretation* component relevant for individual patient decision-making. In other words, the questions related to the tailoring of decisions to individual and/or patient subgroups were linked to the *Evidence* and *Applicability to Patient* items. While questions related to how well-CPGs provide decision support to the stroke clinician were linked to the *Applicability to Target User*, and *Values and Preferences of the Target User* items. In this study, a directed content analysis approach was used (22). Therefore, the interview questions were designed to start with an open-ended question followed by targeted questions about predetermined categories. The rationale and link to the relevant AGREE-REX items for each interview questions are presented in Supplementary Table 1.

The same researcher interviewed all clinicians involved to ensure consistency (VR, PhD candidate). Before the interview, the recruited clinicians were given a 10-min presentation by the interviewer about the rationale for the interview i.e., the need to better understand how stroke experts make individualized decisions about early mobilization post-stroke and how CPGs support this process. As part of the presentation, all expert clinicians were reminded of the current Australian CPG recommendations for early mobilization developed by the Stroke Foundation (23). Demographic data on participants were collected using a brief questionnaire. During the interview, the following topics of interest were examined: the influence of early mobilization evidence on their decision-making, the use of Australian CPGs for decision-making, areas of uncertainty in decision-making, and additional information and parameters required in early mobilization CPG recommendations to support decision-making. The participants were encouraged to discuss and elaborate on other connected areas of discussion if they felt inclined to do so. All participants were interviewed either in-person or via videoconference.

#### Coding and Analysis

The interviews were audio-recorded and transcribed verbatim using a paid transcription program (Amberscript) to facilitate the analysis of data. A *deductive* and *directed thematic content approach* (22) was used based on the main outcomes for the study: clinicians' use of and view on CPGs, specific areas of uncertainty in early mobilization decision-making, and recommendations for improvement of early mobilization CPGs. VR read all transcripts to make initial analytical observations about the data.Transcripts were imported into QSR International NVivo 9 to organize and analyse data using the set of a priori and predefined interview questions. As per the directed thematic content approach, predetermined codes were formed based on the three desired outcomes (24). The strategy for coding involved reading through each transcript and conducting line-by-line coding using the predetermined codes. Subcodes were determined during this process with subsequent analysis. The data that could not be initially coded are identified and analyzed later to determine if they represent a new category or a subcategory of an existing code.

Cross-checking occurred in two ways: firstly, KH double-coded 25% of all transcript across all three coding themes. Differences which occurred were resolved by consensus. Ongoing discussions established trustworthiness and credibility to clarify the interpretation of the data. All subcodes were discussed between VR and KH to determine overlap or divergence in sub-themes within the three broad outcomes. Secondly, KH and VR also randomly selected codes in NVivo to ensure the quotes from the transcripts accurately reflected the theme it was coded to. Descriptive statistics with frequencies and proportions were produced and reported using Stata (version 14.2; StataCorp LP, College Station, TX, USA).

## Results

### Normative Component

#### CPG Selection and Data Extraction

The initial search yielded 57 CPGs. We excluded 39; 31 were published predating the completion of the largest trial on this topic (AVERT); 8 did not include EM recommendations (Supplementary Figure 1, Table 2). The remaining 18 CPGs were from the following geographical regions: Argentina, Australia, Canada, China, Europe, Finland, India, Italy, Korea, Netherlands, Norway, Peru, United Kingdom and United States of America (Supplementary Table 3) (25–27).

#### AGREE-REX Appraisal

**Figure 2:**
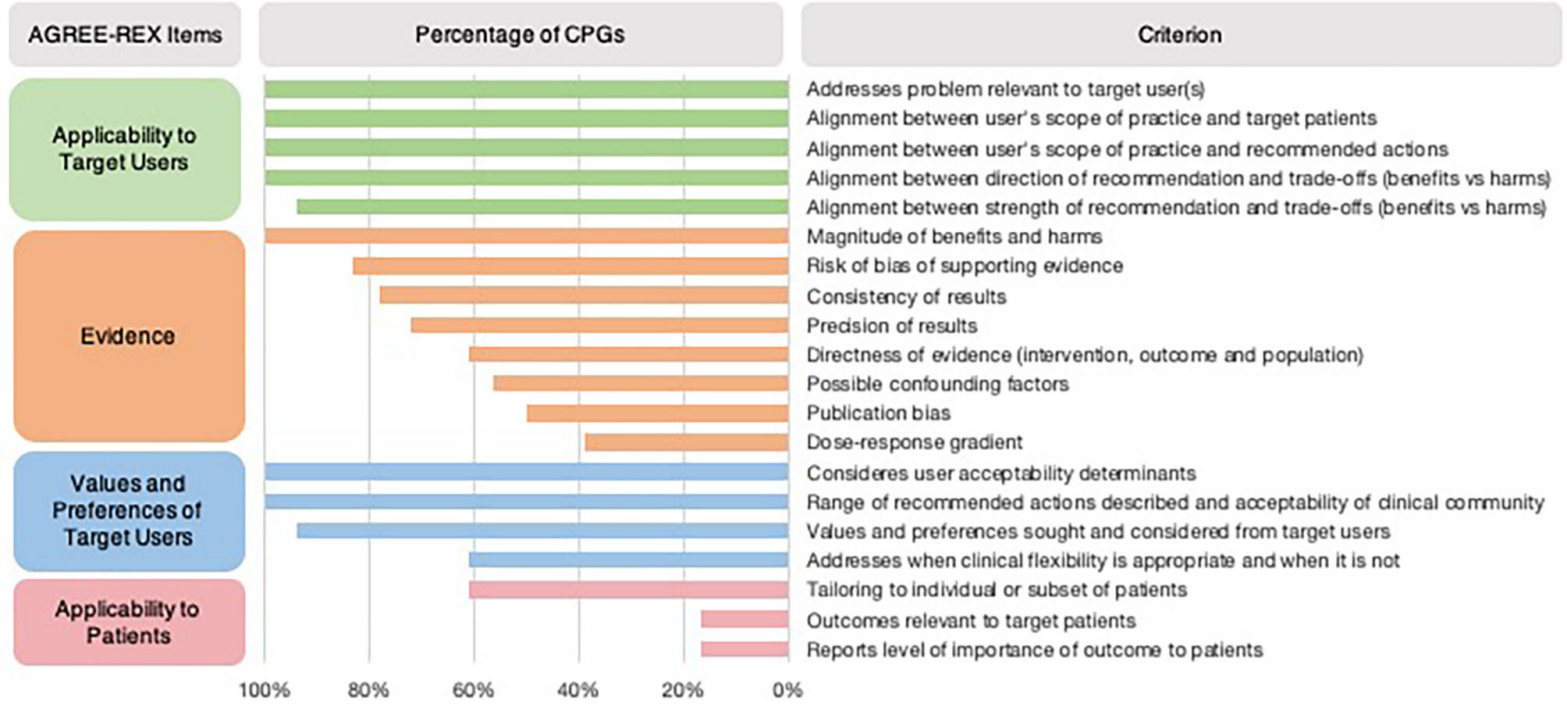
Proportion of CPGs addressing AGREE-REX criteria. Summary of percentage of CPGs addressing each AGREE-REX criterion for the four items.

In most CPGs, *Applicability to the Target User* and *Values and Preferences of the Target User items* were met (Table 1). However, the *Applicability to Target Users* criterion “the guideline differentiates between recommended actions for which clinical flexibility and individual patient tailoring is more appropriate in the decision-making process and those for which it is less appropriate” was only addressed in 61% of CPGs (Figure 2). *Applicability to Patients* was the item with the least number of criteria met by CPGs (Table 1). Within the *Evidence* item, more than 78% of CPGs addressed the magnitude of benefits vs. harms, risk of bias of included studies, and the consistency of results (Figure 2). However, <61% sufficiently addressed the directness of the evidence, confounding factors, publication bias, and dose-response gradient in their recommendations. The *Applicability to Patients* criterion relating to “the tailoring of recommendations to individual patients” was only addressed by 61% of CPGs, while the criteria relating to patient-centered outcomes were addressed by only 17% of CPGs (Figure 2).

**Table 1:** Summary of AGREE-REX item scores.

	**Evidence (%)**	**Applicability to target users (%)**	**Applicability to patients (%)**	**Values and preferences of target users (%)**
Argentina, 2019 (28)	25	100	0	75
Australia, 2019 (27)	100	100	100	100
Canada, 2018 (29)	88	100	33	100
Canada, 2019 (30)	88	100	33	100
China, 2019 (26)	50	100	33	100
Europe, 2018 (31)	50	100	0	75
Finland, 2016 (32)	50	100	0	75
India, 2019 (33)	13	80	33	75
Italy, 2016 (34)	88	100	33	100
Korea, 2017 (35)	50	100	0	75
Netherlands, 2019 (36)	100	100	100	100
Norway, 2017 (37)	100	100	100	100
Peru, 2020 (38)	75	100	33	100
UK, 2016 (39)	63	100	33	100
UK, 2019 (40)	88	100	33	100
USA, 2019 (41)	63	100	0	75
USA, 2016 (42)	63	100	0	75
USA, 2019 (43)	63	100	0	75
All CPGs Median (IQR)	63% (50–88%)	100% (100–100%)	33% (0–33%)	100% (75–100%)

*The proportion of criteria (%) achieved by each CPG and median (IQR) summary scores across all CPGs. Shading emphasizes poor (dark) to good (light) meeting of criteria for each item*.

*CPG, clinical practice guidelines; IQR, interquartile range; UK, United Kingdom; USA, United States of America*.

### Empirical Component

#### Study Participants

Thirty expert stroke clinicians from Australia (Victoria and South Australia) participated in this study; 11 physicians, 11 physiotherapists and eight nurses (Table 2). No clinicians approached declined to participate. On average, clinicians practiced in a stroke context for 14 years (IQR 10-25), 87% are currently practicing, and 74% work in a metropolitan acute stroke care unit.

**Table 2:** Demographic Characteristics of Expert Stroke Clinicians.

**Variables**	**No. (%)**
**Occupation**	
Physician	11 (37%)
Physiotherapist	11 (37%)
Nurse	8 (26%)
**Highest level of education**	
Ph.D.	14 (47%)
Clinical Doctorate	1 (3%)
Masters (clinical)	5 (17%)
Bachelor's Degree	7 (23%)
Graduate Diploma	3 (10%)
**Currently practicing**	
Yes	26 (87%)
No	4 (13%)
**Primary stroke environment**	
Acute stroke unit	22 (74%)
Inpatient rehabilitation	3 (10%)
Outpatient rehabilitation	1 (3%)
Research institute	4 (13%)
**Level of knowledge on early mobilization**	
High, well-informed about evidence	15 (50%)
Average, up to date with evidence	15 (50%)
Low, not up to date with evidence	0 (0%)
Number of years practicing in a stroke context, median (IQR)	14 (10–25)

*IQR, interquartile range*.

#### Use of CPGs as Decision-Support Tools

Out of the total sample, 50% of clinicians perceived their current level of knowledge on early mobilization evidence to be high (Table 2). Only 43% of clinicians indicated that CPGs provide decision-support, 40% expressed their preference to use clinical reasoning with an understanding of current evidence over the use of CPGs. Forty-seven percent of clinicians found current CPGs “too broad or vague,” while 10% thought it was only “useful for junior or non-specialized staff.”

#### Areas of Uncertainty in Decision-Making

**Figure 3:**
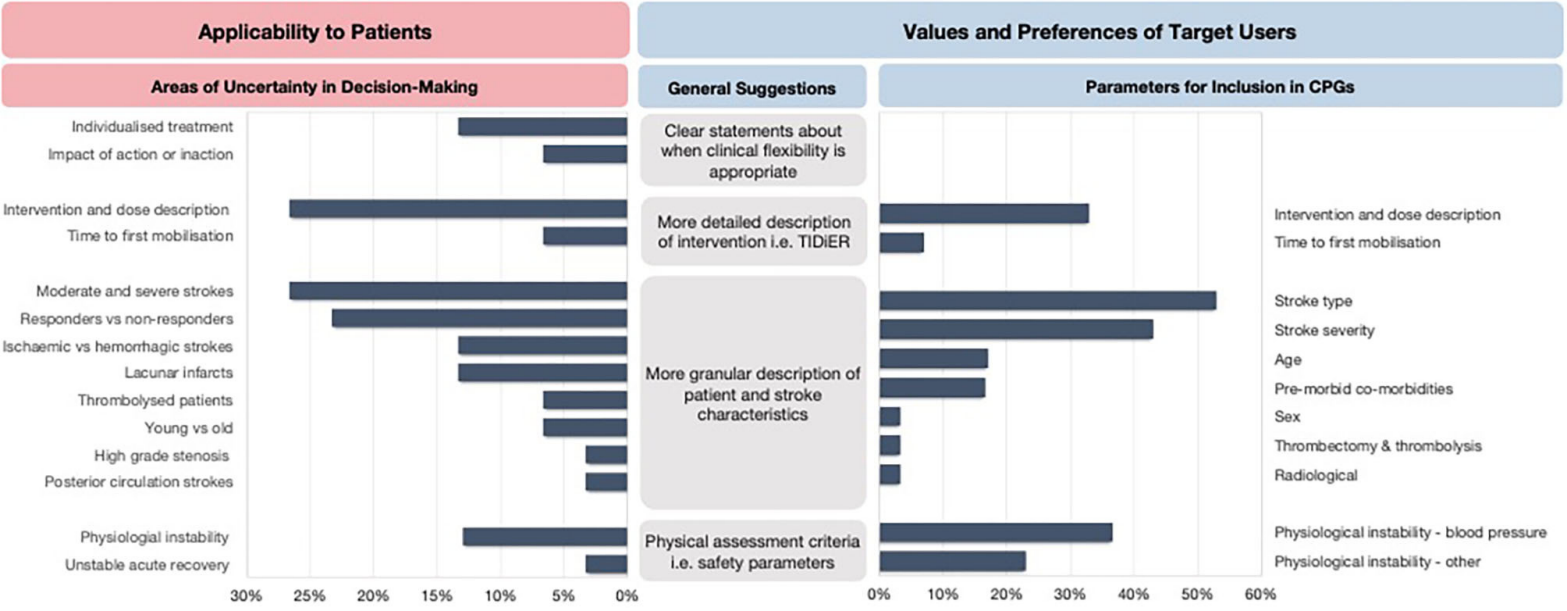
The areas of uncertainty and key recommendations for future CPGs. TIDieR, Template for Intervention Description and Replication.

The areas of uncertainty were generally related to the process of clinical reasoning, the intervention dose, tailoring to specific patient and stroke characteristics and the impact of early mobilization practices on recovery trajectories of patient's post-stroke (Figure 3). Specifically, the three most frequently mentioned areas of uncertainty were related to optimal intervention dose (27%), tailoring early mobilization practices for people with moderate and severe stroke (27%), and the lack of knowledge of potential responders and non-responders to early mobilization practices (23%).

#### Areas of Improvement for Early Mobilization CPGs

In response to the areas of uncertainty, the clinicians expressed the need for CPGs to report more granular descriptions of patient and stroke characteristics to allow for appropriate tailoring of decision-making to an individual or subsets of patients. Other recommendations included clearer statements about when clinical flexibility is appropriate and when it is not, physical assessment criteria to aid decision-making, more detailed descriptions about safety parameters to consider, and a detailed description of the intervention dose. The clinicians also provided specific suggestions for parameters they would like to be reported in early mobilization recommendations (Figure 3).

## Discussion

In this study, we used a novel two-pronged approach to investigate how well current CPGs to support early mobilization decision-making post-stroke for Australian stroke clinicians. The review of early mobilization CPGs demonstrated that almost all CPGs addressed the *Applicability to the Target User* and *Values and Preferences of the Target User* items however, the *Evidence* and *Applicability to Patients* items were not met to the same degree. Therefore, it is not surprising that only 43% of interviewed expert stroke clinicians indicated that CPGs provide decision support, with many often relying on clinical reasoning and individual interpretation of the evidence to select an evidence-based choice of action. Many clinicians (47%) found current CPGs “too broad or vague.” This is an important finding given that specific guidelines can change a physician's decision for the better, while non-specific guidelines can change it for the worse (44). Another important point is that although many Australian stroke experts found Australian CPGs to be “too broad or vague,” the included AGREE-REX item scores for the Australian CPG reached 100%. The discrepancy between our normative and emprical findings may be due to the limited evidence base to effectively support clinical decision-making. There is still a need for more detailed early mobilization research to understand the optimal timing, frequency, and intensity of the intervention. The need for a comprehensive evidence base has obvious implications on how well-CPGs can truly support early mobilization decision-making for a clinician, which may be reflected in our empirical findings. Several areas of uncertainty in decision-making and related suggestions for the improvement of early mobilization CPG recommendations were identified and discussed in more detail below.

The normative component of this study revealed several factors mediating and impeding the use of early mobilization CPGs as decision-support tools. Concerning the *Evidence* item, most CPGs described the magnitude of benefits and harms of early mobilization (mainly informed by AVERT), supporting clinical decision-making by explicitly stating the options for, and implication of, different options. However, many CPGs did not address the possibility of confounding factors or a dose-response gradient despite all CPGs utilizing findings from AVERT, which included safety and intervention parameters. Interestingly, in many CPGs, the exact intervention, population, and outcomes of interest to the clinical problem were not addressed. This was reflected in the interviews with clinicians expressing uncertainty about delivering the optimal dose of early mobilization (exact intervention) to specific subgroups of stroke patients (population) and the impact of early mobilization on a patient's recovery trajectory (outcomes). As such, the findings may highlight potential gaps in the early mobilization evidence base that require future exploration of existing data and the development of new clinical trials to better support evidence-based clinical decision-making.

The *Applicability to Patients* item and the identified areas of uncertainty in clinicians' decision-making exposed several insufficiencies of early mobilization CPGs in providing decision-support. Specifically, many CPGs did not describe how decisions should be tailored to specific patient and stroke characteristics or include patient-centered outcomes. This is supported by the empirical findings from clinicians, which demonstrated uncertainty when making patient-centered decisions. In particular, the main areas of uncertainty included tailoring decisions to potential responders and non-responders, those with moderate and severe strokes, and different stroke types (ischaemic vs. hemorrhagic). By using a novel approach to integrate the newly developed AGREE-REX tool (19, 45) with empirical investigations, this study provides an in-depth understanding about why previous assessment of the methodological quality of CPGs for rehabilitation post-stroke using the AGREE II do not sufficiently address the “applicability” domain (46). It has been suggested that barriers in the uptake of CPGs to support individualized decision-making may be due to the perceived rigidity of CPGs, insufficient clinical flexibility or loss of clinical autonomy (47–50). However, as our normative and empirical data suggests, the absence of granular recommendations is perhaps the motivating reason early mobilization CPGs are underutilized. This is an important barrier that needs to be acknowledged and addressed for the future development of CPGs to support clinicians effectively.

The identified areas of uncertainty directly led to four broad sets of recommendations by stroke clinicians. The recommendations included clearer statements about when clinical flexibility is appropriate, more granular descriptions of patient and stroke characteristics to allow for appropriate tailoring of decision-making, a more detailed description of the intervention dose, and the need for physical assessment criteria including safety parameters. The expert stroke clinicians also suggested specific parameters to be reported in future early mobilization CPGs they deemed necessary when making clinical decisions at an individual patient level. These included consideration of safety parameters and intervention parameters that should be modified to people with different stroke types, stroke severity, age, and comorbidities.

Overall, the apparent need for clear and clinically applicable recommendations directly corresponds to the *Values and Preferences of Target Users* item. While many CPGs sought and considered the values and preferences of clinicians, the criterion requiring specification about when clinical flexibility is appropriate, and when it is less appropriate, was not sufficiently addressed. Clinical flexibility allows “leeway for clinical judgment, patient preferences, and clinically relevant conditions of the delivery system (including necessary equipment and skilled personnel)” (51). Indeed, this is a critical aspect of evidence-based medicine that involves balancing clinical autonomy and reasoning with best available evidence with patients' values and preferences. A decision-support tool needs to specify circumstances when clinical flexibility is necessary to ensure decisions are optimally tailored for specific patients, and when evidence-based standardized care is required. It is clear that clinical decisions about early mobilization post-stroke are complex, multifactorial, and not sufficiently addressed in current CPGs.

This study has some limitations. The use of Google Translate without consultation of clinicians in the country may have been insufficient in contextualizing those recommendations to the local context. While we established the study's scope to enable a comprehensive investigation of the clinical credibility and applicability of current EM CPG using AGREE-REX, we did not assess the methodological rigor and transparency of CPGs. It could be useful to investigate the internal validity of EM CPGs to understand how to improve the overall quality of current CPGs. Another limitation is that we did not include all AGREE-REX items. The unexplored items were related to the values and preferences of patients/populations, values and preferences of policy/decision-makers, purpose, and local application and adoption (19). Consideration of patients and funders' values and perspectives, and alignment across different viewpoints, may enhance the utilization of CPGs (8, 45, 52). Future investigations could adopt our novel two-pronged approach to ensure these normative standards of AGREE-REX are linked with empirical investigation of the different stakeholders (patients, policy or decision makers etc.). All expert stroke clinicians were from Australia, and therefore, our findings are naturally within the scope of an Australian healthcare system and may limit the generalisability of our findings. Nontheless, the use of expert sampling (20) to obtain a multidisciplinary sample of clinical (physicians, physiotherapists, and nurses/nurse practitioners) and academic experts in acute stroke and early mobilization practices allowed the identification of important insufficiencies in decision-support of current CPGs.

The identified lack of specificity, clinical applicability, and adaptability of current CPGs to effectively respond to the heterogeneous clinical stroke context has provided a clear direction for improvement. The four key recommendations for future early mobilization CPGs include more granular descriptions of patient and stroke characteristics for appropriate tailoring of decisions to individual or subgroups of patients; clear statements about when clinical flexibility is appropriate; detailed description of the intervention dose; and physical assessment criteria including safety parameters.

## Data Availability Statement

The raw data supporting the conclusions of this article will be made available by the authors, without undue reservation.

## Ethics Statement

The studies involving human participants were reviewed and approved by The University of Melbourne Human Research Ethics Committee (1851680.1). The patients/participants provided their written informed consent to participate in this study.

## Author Contributions

VR, LC, and JB contributed to the conception and design of the study. VR recruited the participants, conducted the interviews, conducted the systematic review search, collection of the guidelines, organized the database, and wrote the first draft of the manuscript. VR and KH appraised the guidelines and coded the interviews. All authors contributed to manuscript revision, read, and approved the submitted version.

## Conflict of Interest

The authors declare that the research was conducted in the absence of any commercial or financial relationships that could be construed as a potential conflict of interest.
