# Supplementary material for: Early Mobilization After Stroke: Do Clinical Practice Guidelines Support Clinicians' Decision-Making?
Source: Front Neurol. 2021 Feb 5;12:606525. doi: 10.3389/fneur.2021.606525 (PMC7901923; doi:10.3389/fneur.2021.606525)
Supplement: Supplementary file 1 [file Data_Sheet_1.docx]

List of Supplementary Tables

1. Figure 1: PRISMA Flow Diagram
2. Table 1: Interview Questions
3. Table 2: Excluded Early Mobilisation Clinical Practice Guidelines
4. Table 3: Included Early Mobilisation Clinical Practice Guidelines

**Figure 1: PRISMA Flow Diagram**

**
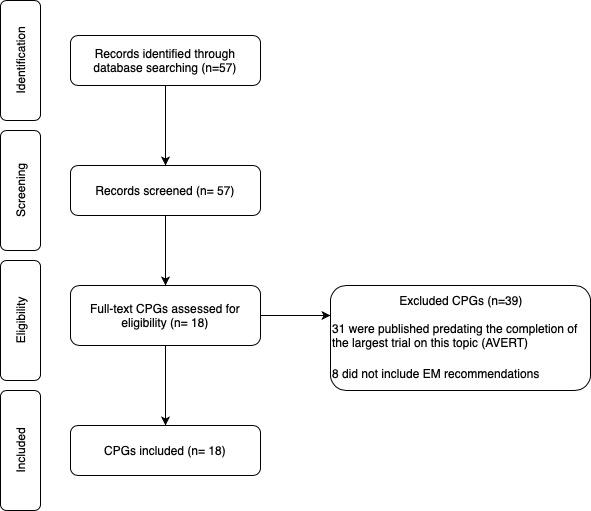
**

**Table 1: Interview Questions**

| **Opinions on Early Mobilisation Clinical Practice Guidelines** |
| --- |
| 1. **Can you talk me through your decision-making process when thinking about mobilising a person early after their stroke?**    - Rationale: To get the clinician to articulate their decision-making process in order to prepare them for the following questions about how CPGs support this process.    - Linked to AGREE-REX: *Evidence* & *Applicability to Patients* |
| 1. **How do clinical practice guidelines influence your decision about early mobilisation?**    - Rationale: To understand if CPGs are utilised by the recruited clinicians and understand how CPGs support decision-making.    - Linked to AGREE-REX: *Applicability to Target Users* |
| 1. **In an ideal world, what would you like guidelines to have that might support your decision-making?**    - Rationale: To understand which aspects of current CPGs are useful and what is needed to better support their decision-making.    - Linked to AGREE-REX: *Values and Preferences of Target Users* |
| 1. **What issues or situations create uncertainty in your decision-making about early mobilisation?**    - Rationale: To understand whether uncertainties in decision-making align with the suggestions to improve CPG recommendations.    - Linked to AGREE-REX:  *Evidence* & *Applicability to Patients* |
| 1. **How do you manage that uncertainty?**    - Rationale: To understand how clinicians negotiate risks vs benefits with the existing knowledge that further evidence about early mobilisation is needed.    - Linked to AGREE-REX:  *Evidence* & *Applicability to Patients* |
| 1. **Do you have any other comments you would like to make?**    - Rationale: To ensure the clinicians have expressed anything else that may be useful in the interpretation of their responses.    - Linked to AGREE-REX: N/A |

**Table 2: Excluded Early Mobilisation Clinical Practice Guidelines**

| **Publication** | **Year** | **Organisation** | **Country** | **Exclusion Reason** | **Current Status** |
| --- | --- | --- | --- | --- | --- |
| Consensus on Diagnosis and Treatment of Acute Ischemic Stroke Council – Argentine Society of Cardiology | 2012 | Argentine Society of Cardiology | Argentina | Pre-2015 | Not updated post-2015 |
| Proposal of Guidelines for Acute Stroke Treatment and Management | 2009 | Belgian Stroke Council | Belgium | Pre-2015 | Not updated post-2015 |
| Diretrizes de Atenção À Reabilitação da Pessoa com Acidente Vascular Cerebral | 2013 | Ministério da Saúde. | Brazil | Pre-2015 | Not updated post-2015 |
| Guidelines for Acute Ischemic Stroke Treatment – Part I and Part 2 | 2012 | Executive Committee from Brazilian Stroke Society and the Scientific Department in Cerebrovascular Diseases of the Brazilian Academy of Neurology | Brazil | Pre-2015 | Not updated post-2015 |
| BEST PRACTICE GUIDELINES FOR THE MANAGEMENT AND REHABILITATION OF STROKE IN THE NORTH WEST REGION OF CAMEROON | 2013 | The NWR Best Practices in Stroke Rehabilitation Group | Cameroon | Pre-2015 | Not updated post-2015 |
| Stroke and Transient Ischemic Attack - Acute and Long-Term Management | 2015 | British Columbia Ministry of Health | Canada | Pre-2015 | Not updated post-2015 |
| Nova Scotia Guidelines for Stroke Care | 2008 | Cardiovascular Health Nova Scotia | Canada | Pre-2015 | Not updated post-2015 |
| Guía de Práctica Clínica Hemorragia subaracnoideaa rotura de aneurismas cerebrales | 2017 | Ministry of Health | Chile | No mobilisation recommendations | NA |
| Guía Clínica Accidente Cerebro Vascular Isquémico, en personas de 15 años y más. | 2013 | Ministry of Health | Chile | Pre-2015 | Not updated post-2015 |
| Guía de práctica clínica de diagnóstico, tratamiento y rehabilitación del episodio agudo del ataque cerebrovascular isquémico en población mayor de 18 años | 2015 | Ministerio de Salud y Protección Social – Colciencias | Colombia | Pre-2015 | Not updated post-2015 |
| Recommendations for Stroke Management 2006 Update | 2006 | Croatian Society for Neurovascular Disorders of Croatian Medical Association, Croatian Stroke Society, and University Department of Neurology, Sestre milosrdnice University Hospital, Reference Center for Neurovascular Disorders of the Croatian Ministry of Health | Croatia | Pre-2015 | Not updated post-2015 |
| Guías clínicas de medicina interna | 2018 | Ministry of Health | El Salvador | No mobilisation recommendations | NA |
| 2016 European Guidelines on cardiovascular disease prevention in clinical practice | 2016 | The Sixth Joint Task Force of the European Society of Cardiology and Other Societies on Cardiovascular Disease Prevention in Clinical Practice (constituted by representatives of 10 societies and by invited experts) Developed with the special contribution of the European Association for Cardiovascular Prevention & Rehabilitation (EACPR) | Europe | No mobilisation recommendations | NA |
| Evidence-based stroke rehabilitation: an expanded guidance document from the European stroke organisation (ESO) guidelines for management of ischaemic stroke and transient ischaemic attack | 2008 | European stroke organisation (ESO) | Europe | Pre-2015 | Not updated post-2015 |
| European Stroke Organisation (ESO) Guidelines for the Management of Spontaneous Intracerebral Hemorrhage | 2014 | European Stroke Organisation (ESO) | Europe | Pre-2015 | Not updated post-2015 |
| Stroke: Early Management (Alert, Pre-hospital Phase, Initial Hospital Phase, Indications for Thrombolysis), Guidelines | 2010 | Haute Autorité de Santé | France | Pre-2015 | Not updated post 2015 |
| Rehabilitation der Mobilität nach Schlaganfall (ReMoS) | 2015 | The German Socienty of Neurorehabilitation | Germany | No mobilisation recommendations | NA |
| Acute therapy of ischemic stroke | 2012 | German Sociecty of Neurology | Germany | Pre-2015 | Not updated post 2015 |
| Manejo Rehabilitativodel ICTUS | 2013 | Instituto Guatemalteco de Seguridad Social | Guatemala | Pre-2015 | Not updated post-2015 |
| Recommendations for the Early Management of Acute Ischemic Stroke: A Consensus Statement for Healthcare Professionals from the Indian Stroke Association | 2018 | Indian Stroke Association | India | No mobilisation recommendations | NA |
| National Clinical Guidelines and Recommendations for the Care of People with Stroke and Transient Ischemic Attack | 2010 | Irish Heart Foundation: Council for Stroke | Ireland | Pre-2015 | Not updated post-2015 |
| Stroke in General | 2011 | Joint Committee on Japanese Guidelines for the Management of Stroke 2009 | Japan | Pre-2015 | Not updated post-2015 |
| Clinical Practice Guidelines, Management of Ischaemic Stroke, 2 Edition 2012 | 2012 | Ministry of Health Malaysia | Malaysia | Pre-2015 | Not updated post-2015 |
| GPC: VIGILANCIA Y PREVENCIÓN SECUNDARIA DE LA ENFERMEDAD VASCULAR CEREBRAL EN EL PRIMER NIVEL DE ATENCIÓN | 2015 | National Center for Technological Excellence in Health | Mexico | Pre-2015 | Not updated post-2015 |
| Management of patients with stroke: Rehabilitation, prevention and management of complications, and discharge planning | 2013 | MOH and health project of MCC, WHO and professional committee of rehabilitation of MOH, Mongolian society of physical & rehabilitation medicine, University of Health Sciences Mongolia (HSUM), the Shastin and State hospital | Mongolia | Pre-2015 | Not updated post-2015 |
| NHG Standard Stroke | 2018 | Dutch College of General Practitioners | Netherlands | Doesn't include AVERT |  |
| New Zealand Clinical Guidelines for Stroke Management | 2010 | Stroke Foundation of New Zealand | New Zealand | Pre-2015 | Stroke foundation guidelines |
| ISCHEMIC STROKE CARE GUIDELINES FOR PAKISTAN | 2009 | Pakistan Society of Neurology | Pakistan | Pre-2015 | Not updated post-2015 |
| Guidelines for the Prevention, Treatment and Rehabilitation of Stroke | 2010 | Stroke Society of the Philippines | Philippines | Pre-2015 | Not updated post-2015 |
| The diagnosis and management of stroke and transient ischaemic attack | 2016 | Ministry of Public Health | Qatar | No mobilisation recommendations | NA |
| Ишемический инсульт и транзиторная ишемическая атака у взрослых | 2015 | Scientific Council of the Ministry of Health of the Russian Federation | Russia | Pre-2015 | Not updated post-2015 |
| 118 Management of patients with stroke: rehabilitation, prevention and management of complications, and discharge planning | 2010 | Scottish Intercollegiate Guidelines Network | Scotland | Pre-2015 | Not updated post-2015 |
| Stroke and transient ischaemic attacks. Assessment, investigation, immediate management and secondary prevention | 2009 | Singapore Ministry of Health | Singapore | Pre-2015 | Not updated post-2015 |
| South African guideline for management of ischaemic stroke and transient ischaemic attack 2010 | 2010 | South African Stroke Society (SASS) | South Africa | Pre-2015 | Not updated post-2015 |
| Management of Stroke |  |  | Sri Lanka | No mobilisation recommendations | NA |
| https://www.socialstyrelsen.se/regler-och-riktlinjer/nationella-riktlinjer/publicerade-riktlinjer/stroke/ | 2020 | Socialstyrelsen | Sweden | No mobilisation recommendations | NA |
| General Principles for the Management of Acute Ischemic Stroke | 2014 | Taiwan Stroke Society | Taiwan | Pre-2015 | Not updated post-2015 |
| Guidelines for Stroke Unit Organization | 2012 | The Neurological Society of Thailand | Thailand | Pre-2015 | Not updated post-2015 |
| ІШЕМІЧНИЙ ІНСУЛЬТ (екстрена, первинна, вторинна (спеціалізована) медична допомога, медична реабілітація) | 2012 | Order of the Ministry of Health | Ukraine | Pre-2015 | Not updated post-2015 |
| Guidelines for the Management of Spontaneous Intracerebral Hemorrhage | 2010 | AHA/ASA | USA | Pre-2015 | Not updated post-2015 |

**Table 3: Included Early Mobilisation Clinical Practice Guidelines**

| **Organisation** | **CPG Document** | **Country** | **Year** | **Recommendation** | **Evidence** |
| --- | --- | --- | --- | --- | --- |
| Sociedad Neurológica Argentina (SNA), Sociedad Argentina de Cardiología (SAC), Sociedad Argentina de Terapia Intensiva (SATI), Sociedad Argentina de Medicina (SAM), Sociedad Argentina de Emergencias (SAE), Colegio Argentino de Neurointervencionitas (CANI), Asociación Argentina de Neurocirujanos (AANC), Sociedad Argentina de Radiología (SAR) y Sociedad Argentina de Geriatría y Gerontología (SAGG). | *Consensus On Acute Ischemic Cerebrovascular Accident* | Argentina | 2019 | It is recommended:  - Early mobilization to prevent complications (Class I, Level of evidence C) Expert Opinion It is not recommended:  - Intense and early mobilization (within 24 hours of the onset of symptoms), as it may reduce the chances of a good prognosis (Class III, Level of evidence B) From one randomised trial | AVERT Group, 2015 |
| Stroke Foundation | [*Clinical Guidelines for Stroke Management*](https://app.magicapp.org/goto/guideline/Kj2R8j/section/nyGJ2j) | Australia/New Zealand | 2017 | 1. For stroke patients, starting intensive out-of-bed activities within 24 hours of stroke onset is not recommended. (Strong recommendation against). i. Baseline stroke severity and stroke type should be considered when deciding when and how much to mobilise after stroke. Intensive out-of-bed activities in the AVERT trial were an average of 6 out-of-bed activities per day (Bernhardt et al. 2015). Patients who are independently mobile should not have their mobility restricted during the early phase after stroke. 2. All stroke patients should commence mobilisation (out-of-bed activity) within 48 hours of stroke onset unless otherwise contraindicated (e.g. receiving end-of-life care). (Strong recommendation) i. Patients with baseline NIHSS scores 4-7 have higher odds of a favourable outcome when they are mobilised more than once per day and spend less than 13.5 minutes per day mobilising with physiotherapy staff (Bernhardt et al. 2016). For patients who have difficulty moving after stroke, an assessment by an appropriately trained health professional as to the most appropriate and safe methods of assisting transfers and out-of-bed activity should be conducted as soon as possible and preferably within 24 hours. 3. For patients with mild and moderate stroke, frequent, short sessions of out-of-bed activity should be provided, but the optimal timing within the 48-hour post-stroke time period is unclear. (Weak recommendation) | 1. Bernhardt et al. 2015 2. Bernhardt et al. 2015; Lynch et al. 2014, Langhorne 2010 3. Bernhardt et al. 2015 |
| Heart and Stroke Foundation, Canada | [*Canadian Stroke Best Practice Recommendations for Acute Stroke Management: Prehospital, Emergency Department, and Acute Inpatient Stroke Care, 6th Edition*](https://journals.sagepub.com/doi/pdf/10.1177/1747493018786616) | Canada | 2018 | *9.4 Mobilization (revised for 2018) Mobilization is defined as ‘‘the process of getting a patient to move in the bed, sit up, stand, and eventually walk.’’* i. All patients admitted to hospital with acute stroke should have an initial assessment, conducted by rehabilitation professionals, as soon as possible after admission [Evidence Level A]. ii. Initial screening and assessment should be commenced within 48 h of admission by rehabilitation professionals in direct contact with the patient [Evidence Level C]. Refer to Canadian Stroke Best Practice Recommendations Stroke Rehabilitation Module for additional recommendations on mobilization following an acute stroke. iii. Rehabilitation therapy should begin as early as possible once the patient is determined to be medically able to participate in active rehabilitation [Evidence Level A]. iv. Frequent, brief, out-of-bed activity involving active sitting, standing, and walking, beginning within 24 h of stroke onset is recommended if there are no contraindications [Evidence Level B]. More intense early sessions are not of more benefit. Clinical judgment should be used. Note: Contraindications to early mobilization include, but are not restricted to, patients who have had an arterial puncture for an interventional procedure, unstable medical conditions, low oxygen saturation, and/or lower limb fracture or injury. | AVERT Group, 2015 and cohort studies and other RCTs |
| Heart and Stroke Foundation, Canada | [*Rehabilitation, Recovery and Community Participation following Stroke Part One: Rehabilitation and Recovery following Stroke Update 2019*](https://www.heartandstroke.ca/-/media/1-stroke-best-practices/rehabilitation-nov2019/2019-csbpr6-rehabrecovery-module-eng-final-dec2019.ashx?rev=d9be6748ea0945368a0733e6b26423ae) | Canada | 2019 | *i. All patients with stroke should receive rehabilitation therapy as early as possible once they are medically stable and able to participate in active rehabilitation [Evidence Level A]. Refer to Section One, Box One: Eligibility and Criteria for Stroke Rehabilitation for more information. ii. Early prolonged mobilization of patients within the first few days after a stroke, especially a severe stroke, is not recommended (Evidence Level A). iii. Earlier mobilization may be reasonable for select patients with acute stroke (for instance people with more mild strokes or transient ischemic attack) but caution is advised, and clinical judgement should be used (Evidence Level C)* | AVERT Group, 2015 and cohort studies and other RCTs |
| Chinese Stroke Association | *Chinese Stroke Association guidelines for clinical management of cerebrovascular disorders: executive summary and 2019 update of clinical management of stroke rehabilitation* | China | 2019 | *Mobilisation within 24 hours after stroke onset is not recommended because it may reduce the possibility for a good outcome at 3 months (Grade III recommendation, Level B evidence).  It is reasonable for patients who had a mild and moderate stroke to do bedside rehabilitation and early rehabilitation away from bed 24 hours after stroke onset. Rehabilitation should be conducted in a step-by-step manner, under supervision if necessary (Grade IIa recommendation, Level A evidence).* | In 2011, the Chinese stroke rehabilitation guidelines recommended that patients who had a stroke should be rehabilitated as soon as stable (stable vital signs, no signs of progression).1 Rehabilitation that starts within 2 weeks following stroke can achieve better outcome.2 A Very Early Rehabilitation Trial (AVERT) explored the effectiveness and safety of super-early activities within 24 hours after stroke onset. The results showed that the super-early activity programme could reduce the benefit at 3 months.3  1. Zhang T, Neurorehabilitation group, Society of Neurology, Chinese Medical Association. Guidelines for stroke rehabilitation in China. Chin J Rehabilitation Theory Pract 2012;04:301–18.Google Scholar 2. Nine Five research group. Study on early rehabilitation of acute stroke. Chin J Rehabil Med 2001;05:266–72.Google Scholar 3. Powers WJ, Rabinstein AA, Ackerson T, et al. 2018 guidelines for the early management of patients with acute ischemic stroke: a guideline for healthcare professionals from the American Heart Association/American Stroke Association. Stroke 2018;49:e46-e110.doi:10.1161/STR.0000000000000158pmid:http://www.ncbi.nlm.nih.gov/pubmed/29367334PubMedGoogle Scholar |
| European Stroke Organisation | [*Consensus statements and recommendations from the ESO Karolinska Stroke Update Conference, Stockholm 11–13 November 2018*](https://journals.sagepub.com/doi/suppl/10.1177/2396987319863606/suppl_file/ESO863606_Supplemental_Material.pdf) | Europe | 2018 | Session 8: Post-stroke early mobilisation Chair: Niaz Ahmed (Stockholm). Speaker: Katharina Sunnerhagen (Goteborg)  Q1: Should we avoid early mobilization after AVERT (A very early rehabilitation trial)?  Recommendations:  1. The evidence point to that early mobilization is safe in stroke patients but should not be too intense (Grade B).  2. A progressive adaptation to activities of daily living, such as going to the toilet with assistance (if needed) or sitting in a chair to eat is fine (Grade A).  3. Patient should be clinically observed and monitored closely and in case they present symptoms noted (Grade C).  4. Early mobilization after a stroke should be adapted to patient’s clinical and neurological situation (Grade C). | Bernhardt et al, 2015  Anderson et al, 2017 |
| Finnish Medical Society Duodecim and the Finnish Neurological Association | [*Cerebral infarction and TIA*](https://www.terveysportti.fi/xmedia/hoi/hoi50051.pdf) | Finland | 2016 | Postural treatment is started immediately to activate the body's senses to promote rehabilitation and prevent body and limb dysfunction.  Early mobilization also reduces the risk of pressure ulcers, pneumonia, deep venous thrombosis and pulmonary embolism [155], but mobilization should not be initiated within the first 24 hours [416]. | European Stroke Organisation (ESO) Executive Committee, 2008  AVERT II, VERITAS, AVERT III  Kaste M, Roine RO. General stroke management and stroke units. In: Stroke, Pathophysiology, Diagnosis and Management. Mohr JP, Choi D, Grotta JC, Weir B, Wolf P (ed.) Philadelphia, Churchill Livingstone, 2004 |
| Directorate General of Health Services Ministry of Health and Family Welfare Government of India | [*Guidelines for Prevention and Management of Stroke*](https://main.mohfw.gov.in/sites/default/files/Guidelines%20for%20Prevention%20and%20Managment%20of%20Stroke.pdf) | India | 2019 | Early mobilization Recommendations 1. All patients should be referred to a physiotherapist/rehabilitation as soon as possible, preferably within 24to 48 hours of admission.  2. Passive full-range-of-motion exercises for paralyzed limbs can be started during the first 24 hours.  3. The patient’s need in relation to moving and handling should be assessed within 48 hours of admission  Early mobility (Mobility between 24-48 hours of stroke) has been associated with improved functional outcomes. To mobilize stroke patients following actions should be taken considering contraindications and termination criteria (Annexure XIV): a. Active and passive range of motion (ROM) exercise should be initiated within 24 hours of onset of stroke. b. ROM exercises should be limited to point of resistance in unconscious patients and range of comfort in conscious patients. c. Bedside sitting and standing can be initiated within first 48 hours of stroke. d. In patients who have undergone angiography through femoral artery, range of motion exercise for hip joint should be deferred for 6 hours post angiography. Monitoring of any bleeding and discomfort should be noted for at least 2 days post angiography.  e. Functional retraining (i.e. participation in bedside mobility, transfers, and activities of daily living) and active participation in self care should be initiated as soon as patient is able to participate. f. In hemorrhagic stroke, mobilization should be delayed till volume of blood gets stabilized for atleast 24 hours. g. Patients with extra ventricular drain (EVD) can be mobilized provided EVD is clamped and secured and intracranial pressure is kept below 20 mm Hg. h. Vital parameters (Heart rate, Blood pressure, Respiratory rate, Temperature), Intracranial pressure if available should be monitored prior to start early mobility, during the session and post interventions. Level 2: Patients who are unable to stand due to poor sensorium or flaccidity, tilt table may be used to make them stand. | Not reported |
| SPREADS – Stroke Prevention and Educational Awareness Diffusion | [*Brain stroke: Italian guidelines for prevention and treatment Recommendations and Synthesis*](http://www.iso-spread.it/index.php?azione=capitoli#end) | Italy | 2016 | Recommendation 14.2.e Strong in favor In stroke patients it is recommended to carry out interventions as early as the first 24 hours mobilization and rehabilitation activities (moderate intensity), if they do not exist contraindications to the program.   Recommendation 14.2.f Strong against In the first 24 hours after a stroke, mobilization interventions are not recommended intensive with early verticalization, particularly in severe strokes and strokes bleeding. | AVERT III |
| Korea Society for Neurorehabilitation | [*Clinical Practice Guideline for Stroke Rehabilitation in Korea 2016*](applewebdata://A09F2409-172E-4A13-BAC0-5BBEDA72B4CE/../Downloads/bn-10-e11%20(1).pdf) | Korea | 2017 | 1-2-1. It is strongly recommended that rehabilitation of acute stroke patients be started as soon as possible when medically stable. (Recommended level A, evidence level 1 ++) 1-2-2. Patients with acute stroke should begin rehabilitation within 72 hours after stroke. (Recommended level B, evidence level 1+)  2-1-1. Patients should move from bed early unless there are contraindications, and it is strongly recommended to carry out wheelchair movements and standing walking. (Recommended level A, evidence level 1 ++) 2-1-2. It is not recommended that all stroke patients undergo high intensity gait training uniformly within 24 hours of onset. (Recommended level B, evidence level 1+) 2-1-3. Stroke patients with movement disorders should consider comprehensive exercise recovery programs early, unless contraindicated. (Recommended level GPP) | Several trials including AVERT II and III, Sundseth, VERITAS |
| Federation of Medical Specialists (Dutch Association for Neurology) | [*Cerebral infarction and bleeding*](https://richtlijnendatabase.nl/richtlijn/herseninfarct_en_hersenbloeding/revalidatie_na_herseninfarct_-bloeding.html#verantwoording) | Netherlands | 2019 | Mobilize patients with severe cerebral infarction or major bleeding (NIHSS >16) during the first days after a cerebral infarction briefly (no more than 10 consecutive minutes) and frequently (at least 2 to 3 times a day). Mobilize the remaining patients with cerebral hemorrhage or cerebral infarction to the best of their ability.   High GRADE There is strong evidence that patients should be able to be mobilized. There is no evidence that secondary complications can be prevented within 24 hours by early mobilization. There is no evidence that by mobilizing patients early, neurological recovery, walking ability and ADL independence can be favorably influenced after three months. There is no evidence that early mobilization has a beneficial effect on the duration of admission to a stroke unit.  Mediocre GRADE There is indirect evidence that early mobilization within 24 hours after a cerebral infarction or stroke may be detrimental to patients with stroke or severe neurological failure (NIHSS> 16) when admitted to a stroke unit. | References AVERT III, AVERT II Bernhardt, 2004 Bernhardt, 2009 (Cochrane Review)  Cumming, 2008 Cumming, 2011 Otterman, 2012 Van Wijk, 2012 |
| Norwegian Directorate of Health | [*National professional guideline for treatment and rehabilitation of stroke*](https://www.helsedirektoratet.no/retningslinjer/hjerneslag/akuttfasen-undersokelse-og-behandling-ved-hjerneslag/komplikasjoner-forebygging-og-behandling) | Norway | 2017 | For patients with stroke, early mobilization is recommended. SIGN B 2a. Frequent mobilization of short duration in the early phase is suggested. (Strong Recommendation).  Practical - this is how the recommendation can be followed Bed rental disposes for immobilization-related complications and early mobilization in stroke unit seem to reduce such complication, and early mobilization is therefore recommended for most patients with acute stroke. [170] ; [171]. However, very early mobilization <24 hours combined with intensive exercise can have negative effects [169]. Early mobilization should therefore be individualized and adapted to the individual patient.  Time of mobilization Most patients with mild stroke (National Institute of Health Stroke Scale (NIHSS) 0-5)) who have stable or decreasing symptoms and stable BT can be mobilized within the first 24 hours after onset. For patients with moderate to severe symptoms (NIHSS> 5) and stable BT, initial mobilization should be performed between 24-48 hours after onset. In unstable patients with fluctuating or increasing symptoms and unstable BT, mobilization should usually be postponed until the patient is stable.  First mobilization Prior to initial mobilization, the patient should be well hydrated (saline solution may be given 500-1000 ml intravenously). At initial mobilization, BT should also be measured before starting and after 15 minutes. For BT falls> 20mmHg or increasing symptoms including dizziness, mobilization should be interrupted. Patients should follow an "out-of-bed protocol" adapted to the individual, but with the aim of regaining standing and walking function and ADL function. During the first 24-48 hours, mobilization should consist of short periods several times daily (4-6) out of bed - initially of duration 15-20 minutes and gradually increasing if the patient's condition, including BT, is stable.  Content of early mobilization The mobilization consists of the patient getting into a sitting, standing or walking position, depending on the level of function. Exercise should be task-related and include daily activities such as transfer, personal care, eating, dressing and undressing and toilet visits. Patients need motivation for self-activity, and appropriate guidance and help when needed. Everyone in the multidisciplinary team should help to mobilize the patient early, initially with short periods out of bed, and during observation.  The amount of exercise and duration of exercise periods out of bed seems to be of greater importance than the time of start of mobilization, at least in the time period up to 48 hours after onset. Therefore, when designing the recommendation and the practical advice, one has chosen to place considerable emphasis on the dose; frequency and duration based on clinical experience and analyzes from the AVERT study [168]. In addition, emphasis has been placed on the documentation available from stroke unit studies indicating that long-term bed rest is negative [1] . | AVERT III Govan, et al, 2007 Indredavik et al, 1999 Legg et al, 2007 Sundseth et al, 2012 Stroke Unit Trialists'Collaboration, 2013 (Cochrane Review)  Holte al, 2016. |
| Este documento debe ser citado como: “Instituto de Evaluación de Tecnologías en Salud e Investigación. Guía de Práctica Clínica para el diagnóstico y tratamiento de la etapa aguda del Ataque CerebrovascularIsquémico: Guía en Versión Corta. Lima: EsSalud; 2018” | GUÍA DE PRÁCTICA CLÍNICAPARA EL DIAGNÓSTICO Y TRATAMIENTO DEL PACIENTE CON ATAQUE CEREBROVASCULAR ISQUÉMICO (ACVi) | Peru | 2018 | Disability (early start of rehabilitation therapy at 72 hours) The Colombian Guide uses Lynch's SR (142), which identifies clinical trials and cohorts without actually carrying out meta-analyzes, indicating that in two cohort studies the effects of starting physical rehabilitation in the first 3 days (72 hours) were compared with 4 or more days, said intervention improved the probability of reducing disability by 15.3% (p <0.001), very low quality of evidence.  Mortality (early start of mobilization at 24 hours) The early mobilization of patients after a stroke (within 24 hours) does not show significant differences in mortality at three months compared to late mobilization (4 studies, n = 2446, RR 1.21, 95% CI 0.76 to 1.65, I2 0%, p = 0.54, quality of evidence moderate).  Disability (early start of mobilization at 24 hours) The early mobilization of patients after a stroke (within 24 hours) does not show significant differences in disability (Rankin 0 to 2) at three months compared to late mobilization (5 studies, n = 2373, RR 0.80, 95% CI 0.58 to 1.02, I2 45%, p = 0.12, low quality of evidence).  It is suggested that stroke patients with some degree of disability include them in physical rehabilitation programs. It is suggested to start physical rehabilitation within 72 hours and mobilization of the patient within 24 hours taking into account the patient's condition and hemodynamic status. RConditional in favorLow (⊕⊕⊝⊝) | Li et al, 2018 |
| Royal College of Physicians Intercollegiate Stroke Working Party | [*Royal College of Physicians 2016 National clinical guideline for stroke.*](https://www.strokeaudit.org/SupportFiles/Documents/Guidelines/2016-National-Clinical-Guideline-for-Stroke-5t-(1).aspx) | UK | 2016 | *Early mobilisation  Immobility and/or bed rest are well-documented to have detrimental effects on hospital patients in general. Early mobilisation (e.g. activities such as sitting out of bed, transfers, standing and walking) aims to minimise the risk of the complications of immobility and improve functional recovery.  A Patients with difficulty moving after stroke should be assessed as soon as possible within the first 24 hours of onset by an appropriately trained healthcare professional to determine the most appropriate and safe methods of transfer and mobilisation.  B Patients with difficulty moving early after stroke who are medically stable should be offered frequent, short daily mobilisations (sitting out of bed, standing or walking) by appropriately trained staff with access to appropriate equipment, typically beginning between 24 and 48 hours of stroke onset. Mobilisation within 24 hours of onset should only be for patients who require little or no assistance to mobilise.* | A Working Party consensus B AVERT Trial Collaboration group 2015; Bernhardt et al, 2016 |
| National Institute for Health and Clinical Excellence | [*Stroke and transient ischaemic attack in over 16s: diagnosis and initial management*](https://www.nice.org.uk/guidance/ng128/chapter/Recommendations#optimal-positioning-and-early-mobilisation-for-people-with-acute-stroke) | UK | 2019 | *Early mobilisation 1.7.2 Help people with acute stroke to sit out of bed, stand or walk as soon as their clinical condition permits as part of an active management programme in a specialist stroke unit. [2019] 1.7.3 If people need help to sit out of bed, stand or walk, do not offer high-intensity mobilisation in the first 24 hours after symptom onset. [2019]* | <https://www.nice.org.uk/guidance/ng128/evidence/f-very-early-mobilisation-pdf-6777399571> |
| American Heart Association/American Stroke Association | [*Guidelines for the Early Management of Patients With Acute Ischemic Stroke: 2019 Update to the 2018 Guidelines for the Early Management of Acute Ischemic Stroke: A Guideline for Healthcare Professionals From the American Heart Association/American Stroke Association*](https://www.ahajournals.org/doi/full/10.1161/STR.0000000000000211) | USA | 2019 | *High-dose, very early mobilization within 24 hours of stroke onset should not be performed because it can reduce the odds of a favorable outcome at 3 months. (III: Harm; B-R)* | Supplementary: Table LXVIII. Randomized Clinical Trials of Mobility Intervention included: SEVEL, 2016; Morreale, 2016; AVERT III, 2015 |
| American Heart Association/American Stroke Associa tion | [*Guidelines for Adult Stroke Rehabilitation and Recovery*](https://www.ahajournals.org/doi/pdf/10.1161/STR.0000000000000098) | USA | 2016 | *High-dose, very early mobilization within 24 hours of stroke onset can reduce the odds of a favorable outcome at 3 months and is not recommended. (III; A)* | AVERT II, AVERT III |
| Department of Veterans Affairs and Department of Defense | *VA/DoD Clinical Practice Guideline for the Management of Stroke Rehabilitation* | USA | 2019 | *There is insufficient evidence to recommend for or against implementing very early mobilization (within 24-48 hours) to improve functional outcomes. (Neither for nor against \| Reviewed, New-added)* | Li Z, Zhang X, Wang K, Wen J. Effects of early mobilization after acute stroke: A meta-analysis of randomized control trials. J Stroke Cerebrovasc Dis. May 2018;27(5):1326-1337. PMID: 29373228.  Xu T, Yu X, Ou S, Liu X, Yuan J, Chen Y. Efficacy and safety of very early mobilization in patients with acute stroke: A systematic review and meta-analysis. Sci Rep. Jul 26 2017;7(1):6550. PMID: 28747763.  Langhorne P, Wu O, Rodgers H, Ashburn A, Bernhardt J. A Very Early Rehabilitation Trial after stroke (AVERT): A phase iii, multicentre, randomised controlled trial. Health Technol Assess. Sep 2017;21(54):1-120. PMID: 28967376. |
